# Bad faith, medical education, and post-truth

**DOI:** 10.1007/s40037-017-0394-5

**Published:** 2017-12-18

**Authors:** Alan Bleakley

**Affiliations:** Poldown Cottage, Escalls Cliff, Sennen, Penzance, Cornwall UK

Kaylee Eady and Katherine Moreau’s [1] article in this issue raises a number of complex, interconnected issues. It is not uncommon for research to report people’s perceptions about what they might do, rather than what they actually do. Indeed, this perspective is noted as a limitation to their study by the authors themselves:

This study described residents’ and physician-educators’ *perceptions* of how they would use parent feedback, and does not report descriptions of their *actual use* of this feedback.’ (My emphases).

The choice to not study ‘actual use’ of feedback in clinical practice seems to me to be a flaw in research design, an inevitable consequence of which is that the study report is peppered with use of the conditional tense—‘would’ and ‘could’. In situating the study’s results in the world of the possible and not the actual, the authors mirror the common medical education practice of learning through simulation.

*That* clinicians and medical educators would use feedback from patients and family members in a paediatric context is assumed—collaborative decision-making is surely a sine qua non for both medicine and medical education in an era that strives for democratic practices. *How* such feedback might be used promises a new level of insight, but readers are instead offered a foregone conclusion rather than a research revelation:

to: (a) provide additional direct observations of residents’ performances, (b) teach and coach residents, (c) assess residents’ overall performance and progression, and (d) encourage resident self-assessment and behaviour change.

However, as I reflected further on these ‘as if’ conditionals—the ‘woulds’ and ‘coulds’—what emerged from the surface data was a deeper notion: that medical education is caught in a double bind. On the one hand, definitive answers or certainty are craved, yet the reality of medicine is that it inhabits the ‘swampy lowlands’ [2] of practice, often dealing with multiple, complex and messy presenting symptoms, in other words uncertainty, uniqueness and values conflict. In such territory, the only means of survival for both doctor and patient is to co-educate for tolerance of ambiguity, perhaps medical education’s key project. For a discipline that seeks to be grounded in science and evidence, medical education is actually largely speculative, soaked in metaphor, and is also strangely satisfied with pedagogical underachievement (where medicine is typically framed as attracting high achievers). For example, why do we seek ‘competence’ when the word literally means ‘good enough’—pedagogy is surely more complex than this? Why do we frame patients as ‘problems’ (problem-based learning)? Why do we consistently describe medical education as ‘training’, where the root of the word means ‘to trail behind’, surely not a productive image of the relationship between teachers and learners? And why do we ‘train’ clinical ‘skills’ instrumentally, when clinical encounters are typically laced with complex feelings?

As introduced above, as a resonance with its research design (‘as if’ rather than ‘this is’) Eady and Moreau’s article reminds me of the central place of simulation and role-play in relation to the development of identity in medicine. Because of patient safety issues, medical students have to learn clinical skills (including invasive procedures) and intimate examinations in simulated conditions. This makes sense. What does not immediately make sense is that this ‘safe’ practice is extended—using actor patients—to communication and teamwork ‘skills’ (can communication and teamwork really be reduced to an instrumental set of ‘skills’?), including psychiatric examinations. Students then have to enter a fantasy world of role-play (with a prepared script) just as the actor patient (with his or her prepared script) must. Medical students might think, from working in simulation with ‘standardized’ actor patients (i. e. working from standardized scripts), that patient encounters can be readily boxed and shelved—the Enlightenment fantasy of classification of knowledge as a form of sovereign power and control [3]. This confuses the naked diagnostic encounter (often dealing with differentials) with the actual flesh-and-blood clinical encounter.

In their virtual imprisonment in the medical school’s ‘clinical and communication suites’—that supposedly simulate clinical settings but actually stereotype them—students may, in a sense, never mature. ‘Medical student’ identities—also the ‘subjects’ of surveillance—can become suspended in aspic rather than progressing to the status of ‘trainee doctor’, where natural humanity (and humility) may serve as a better basis for communication once in the swampy lowlands of real clinics with idiosyncratic patients. Here is an existential dilemma for medical students—held between simulation and reality they may be prone to developing what Jean-Paul Sartre referred to as ‘bad faith’—self-deception and inauthenticity. The development of the identity of the self-aware trainee doctor is frustrated as the institution of the medical school—its senior professionals and educators—retain and choose how to (re)distribute the capital of emotional, professional and clinical knowledge that would turn a supplicant medical student into an authentic proto-professional and trainee doctor.

By reading Eady and Moreau’s paper for its metaphorical yield, I arrived at a less hostile position towards its persistent resort to the conditional tense. The urgent need for medicine to democratize conjures up the fiction that democracy is an achievable finished state, an end point. Jacques Derrida [4] eloquently speaks of a conditional democracy, process not product, a ‘democracy-to-come’ or horizon. Medical education—as an education for democratic habits—must then inhabit the shifting ground of indeterminacy, uniqueness and values conflict, and must generate non-authoritarian identities grounded in tolerance of ambiguity. Such pedagogy can resist Sartre’s ‘bad faith’ in an embrace of authenticity. Here, the bubble of self-deception engendered by simulation and role-play is burst. Emergent democracy in medicine must flourish within the climate set by the conditionals of ‘possibly’, ‘maybe’, ‘let’s see’. As Claude Lefort [5] suggests: ‘Democracy is a form of society in which persons consent to live under the stress of uncertainty’.

The subtext to Eady and Moreau’s paper goes beyond simulation to offer an implicit essay on the simulacrum. Simulation is a copy of something that exists (for example, where reality offers unacceptable risk or danger). A simulacrum is a copy where there is no original. Learning communication skills as supposed simulation enters this territory because there is no ‘original’ blueprint for what is ‘good’ communication, where context (the swampy lowland) is continually changing underfoot. In the ‘post truth’ age, the simulacrum does not conceal the truth. Rather, the truth conceals that there is no truth; or, the simulacrum is true. We are all now familiar with the world of the simulacrum from the Presidency of Donald Trump. Where Trump claims authenticity for blatant untruths, and badges authentic reporting as ‘fake news’, we inhabit a world in which inauthenticity becomes a style of life. From Lewis Carroll’s *Alice in Wonderland*: ‘the dodo says the hatter tells lies/the hatter says the march hare tells lies/the march hare says that both the dodo and the hatter tell lies.’ Who, then, is telling the truth?
